# Supplementary material for: Splice-Junction-Based Mapping of Alternative Isoforms in the Human Proteome
Source: Cell Rep. Author manuscript; Available in PMC 2020 Jan 15. (PMC6961840; doi:10.1016/j.celrep.2019.11.026)
Supplement: 2 [file NIHMS1546469-supplement-2.zip › DF1/PXD006675/LeftCoronaryArtery/LeftCoronaryArtery_18_HDLBP_NLANIAEVEVSIPAK.pdf]

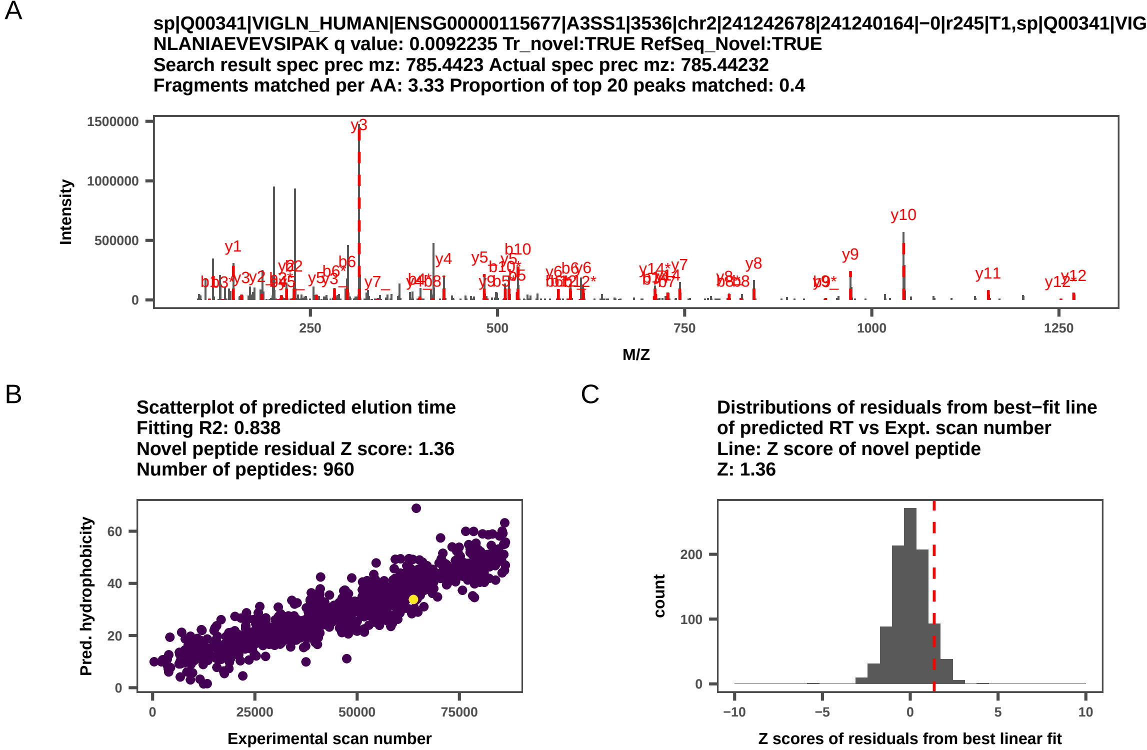
A
sp|Q00341|VIGLN_HUMAN|ENSG00000115677|A3SS1|3536|chr2|241242678|241240164|−0|r245|T1,sp|Q00341|VIG
NLANIAEVEVSIPAK q value: 0.0092235 Tr_novel:TRUE RefSeq_Novel:TRUE
Search result spec prec mz: 785.4423 Actual spec prec mz: 785.44232
Fragments matched per AA: 3.33 Proportion of top 20 peaks matched: 0.4
Intensity
1500000
1000000
500000
0
y3
y1
b6
y4
y5
b10
y7
y8
y9
y10
y11
y12
250
500
750
1000
1250
M/Z
B
Scatterplot of predicted elution time
Fitting R2: 0.838
Novel peptide residual Z score: 1.36
Number of peptides: 960
Pred. hydrophobicity
60
40
20
0
0
25000
50000
75000
Experimental scan number
C
Distributions of residuals from best−fit line
of predicted RT vs Expt. scan number
Line: Z score of novel peptide
Z: 1.36
count
200
100
0
−10
−5
0
5
10
Z scores of residuals from best linear fit
